# Laryngotomy, after Apparent Death—Recovery

**Published:** 1856-10

**Authors:** George Amerman

**Affiliations:** Late House Surgeon of Bellevue Hospital


THE NORTH-WESTERN

MEDICAL AND SURGICAL JOURNAL.

(NEW SERIES.)

VOL. V. OCTOBER, 1856. NO. 10.

### ORIGINAL COMMUNICATIONS.

ARTICLE I.—*Laryngotomy, after Apparent Death—Recovery.* By GEO. AMERMAN, M.D., late House Surgeon of Bellevue Hospital.

Mary Smith, aged 25, widow, born in Canada; parents both living, and in good health. During her residence in Canada she enjoyed uniform good health, except having had those diseases incident to childhood, and one or two attacks of intermittent fever. At the age of fifteen she was married; one year after she was delivered of her first child, which was perfectly healthy and is still living. Two years after her first confinement, she gave birth to her second child, which was, also, at the time of birth in good health, but died four months after with some affection of the bowels. She then left her husband and removed to the city of New York. Two years after her arrival she contracted primary syphilis, which was cured in six weeks. One year afterwards she began to suffer from secondary symptoms—sore throat and eyes. Two years ago she gave birth to her third and last child, which, unlike the two preceding, was sickly and puny, and died at the age of thirteen months. Three months ago the difficulty of the throat greatly increased, and she was also attacked with rupia. She applied to a physician, but, owing to the exposure she was obliged to undergo, she failed to obtain relief, and was admitted into Bellevue Hospital, April 10th, 1856. At the time of her admission, she had large bullæ over the whole upper part of the body and head. The throat was not carefully examined; and I am unable to say whether or not it was ulcerated. She had no dyspnœa or pain; her pulse was good, but her condition indicated an impaired constitution and a general syphilitic cachexia. She was ordered small doses of hyd. bi-chlo., with the tinct. cinch. co., good diet, and anodynes at night. About five hours after my visit, I was hastily summoned to her by the nurse, who told me she was dying. I found her sitting up in bed, unable to speak and gasping for breath; her lips and ends of her fingers were livid, and the veins of the neck distended. The dyspnœa was so severe that it seemed to threaten immediate suffocation. On examining the throat, nothing was perceptible, except a general redness of the fauces, which was most intense on the anterior surface of the epiglottis. Pressure over the larynx caused some pain. Inspiration was more difficult than expiration. Physical examination of the chest revealed nothing abnormal. The air entered the lungs and was resonant throughout. It was thought expedient to try scarifications, which, however, proved useless. An emetic of zinci sulphas was next given, but without producing emesis. Her dyspnœa had now become so alarmingly urgent, that it was decided to open the air passages. Laryngotomy was the operation proposed, as the obstruction was thought to exist in the larynx—probably at the vocal cords. Some time necessarily elapsed in preparing the instruments, &c., prior to the operation; and, just as I was about to make the first incision, the patient gave a gasp, and apparently expired. All respiration, or efforts to respire, ceased. The pulse, which had been before extremely feeble and rapid, now became more so; and, in half a minute after she ceased to breathe, her heart stopped pulsating. At the moment respiration ceased, I proceeded rapidly with the operation, and in one and a half minutes succeeded in introducing the tube. Artificial respiration was resorted to, and in less than a minute (between one-half and one minute) natural respiration commenced. I think there was an absence of the respiration for at least two minutes, and of the pulse for at least one and a half minutes. The respiration ceased first and was first restored. When the patient was fully resuscitated, her pulse, though feeble, was regular and much slower, her respiration perfectly easy, and she sank into a quiet and refreshing sleep. Some trachitis followed the operation; but her recovery was as rapid as could have been expected. She wore the tube for twelve days, when it was removed and the wound allowed to close. Her rupia was treated with alterative doses of hyd. bi-chlo. and tinct. cinch. co.; but it seemed to have very little good effect, and, instead, large doses of iod. potassa were substituted, which acted promptly and beneficially. The large scabs became detached, and slowly separated, leaving a healthy ulcer underneath, which rapidly healed. Her recovery was complete. The wound in the throat closed, and respiration through the natural passages became free and easy.

### REMARKS.

During my residence in Bellevue Hospital, laryngotomy was performed four times for syphilitic disease of the throat, and in all the cases the operation was successful. Two of them left the Institution entirely cured. In both, the tube was worn for a shorter time than one month, when they were able to breathe easily through the natural passages, all symptoms of disease of the throat having disappeared. The third case died, eight months after the operation, from lupus, which had extended to the base of the scull and excited secondary arachnitis, which was the immediate cause of death. In this case the tube was never removed. The fourth case was the one whose history I have given above. It was perfectly successful. The tube was worn for twelve days, when all signs of disease in the throat disappeared. This case is also interesting in two other particulars:—*First,* As showing the necessity of the operation; and, *Secondly*, the complete relief afforded by it. So far as these cases go, they point to the conclusion already arrived at by some surgeons, that, in cases of acute disease supervening on chronic syphilitic affections of the throat, the obstruction is situated in the larynx, and that laryngotomy affords relief, and hence, being an easier operation than tracheotomy, should be performed.

